# Plan and operations of the 10th Korea National Health and Nutrition Examination Survey (2025–2027)

**DOI:** 10.4178/epih.e2026001

**Published:** 2026-01-02

**Authors:** Sun-Ja Kim, Sihyun Park, Sunja Kim, Suyeon Park, Yoonjung Kim, Yunjung Choi, Sungha Yun, Kyungwon Oh

**Affiliations:** Division of Health and Nutrition Survey and Analysis, Bureau of Chronic Disease Prevention and Control, Korea Disease Control and Prevention Agency, Cheongju, Korea

**Keywords:** Korea National Health and Nutrition Examination Survey, Health policy, Health surveys, Nutrition surveys, Longitudinal studies

## Abstract

The Korea National Health and Nutrition Examination Survey (KNHANES) is a national health survey mandated by Article 16 of the National Health Promotion Act to assess the health and nutritional status of the Korean population. Over the past 2 decades, the survey has continuously introduced timely survey components while strengthening its survey methodology and operational systems to enhance both the policy relevance and scientific utility of its findings. The 10th KNHANES (2025–2027) preserves the statistical representativeness of its sampling design while expanding the use of web-based self-administered questionnaires to improve participant convenience. In response to Korea’s transition into a super-aged society, the 10th KNHANES incorporates enhanced older-adult health surveys, including osteoporosis assessment and older adults’ life functioning, and newly introduces items addressing social support as part of the social determinants of health. In addition, a longitudinal follow-up survey system has been established to monitor long-term changes in population health. Collectively, these changes are expected to improve understanding of aging-related health issues, support evidence-based national health policy development, and expand the applicability of KNHANES data for academic research.

## INTRODUCTION

The Korea National Health and Nutrition Examination Survey (KNHANES), conducted under Article 16 of the National Health Promotion Act, assesses the health and nutritional status of the Korean population, supports the development and evaluation of national health policies, and provides health indicators to international organizations such as the World Health Organization (WHO) and the Organization for Economic Cooperation and Development (OECD) [1-8]. From 1998 to 2005, the survey was conducted every 3 years as a short-term survey. To improve the timeliness of statistical outputs, reduce seasonal bias, and strengthen survey expertise and continuity, the system was converted to a year-round rolling survey (January–December, 48 weeks) with dedicated field survey teams beginning with the fourth KNHANES (2007–2009) [9,10]. Since that transition, the survey has been conducted annually and was completed without interruption, even during the coronavirus disease 2019 (COVID-19) pandemic.

The survey targets all residents of Korea aged ≥1 year. Approximately 400 survey items encompass health behaviors, major chronic diseases, dietary behaviors, and food and nutrient intake. Data are collected through health examinations, face-to-face health interviews, and self-administered questionnaires administered at Mobile Examination Centers (MECs) (Supplementary Material 1). Systematic quality-control procedures are implemented to ensure the accuracy and reliability of the collected data. Survey results are reviewed by the national health survey advisory committee (advisory committee) and published annually, and the corresponding microdata are made publicly available [11]. More detailed information on KNHANES is available on the official website (https://knhanes.kdca.go.kr).

This paper describes the major updates and operational improvements of the 10th KNHANES (2025–2027) and presents the rationale for these changes, along with their implications for future policy and research applications.

## PLAN FOR THE 10TH KOREA NATIONAL HEALTH AND NUTRITION EXAMINATION SURVEY (2025–2027)

This section summarizes the main components of the 10th KNHANES, including the target population, survey contents, data collection procedures, field operations, data utilization, the follow-up survey, and international collaboration. Key changes are presented in Table 1.

### Survey population

Since the introduction of the rolling survey system in the fourth KNHANES (2007–2009), the overall sample size has remained largely unchanged, thereby maintaining a scale comparable to that of previous survey cycles. The sampling design employs a 2-stage stratified cluster sampling method, in which enumeration districts (EDs) serve as the primary sampling units (PSUs) and households serve as the secondary sampling units. The sampling frame is based on the most recent 2022 Population and Housing Census data. To select 25 households per ED, 395,297 basic census EDs, each averaging approximately 60 households, were merged into about 150,532 combined EDs, with each combined ED comprising roughly 150 households. To ensure representativeness and estimation accuracy, the sampling frame was stratified by 17 metropolitan cities and provinces, housing type (general housing and apartments), and urban or rural classification. Additional implicit stratification variables included gender ratio, age distribution, housing size, age of the household head, and the proportion of single-person households (Supplementary Material 2). Sample allocation was proportionally distributed across metropolitan cities and provinces, housing types, and urban or rural areas. In regions such as Sejong and Jeju, where securing a minimum sample size was necessary, at least 2 EDs per stratum were allocated prior to proportional allocation. For example, in Seoul’s general housing stratum, 16,239 EDs were grouped into 3 implicit strata, sorted, and 19 EDs were selected proportionally [12]. Within each sampled ED, 25 households were selected from eligible households, excluding collective living facilities (e.g., nursing homes, military barracks, prisons) and foreign households.

### Survey contents

The 10th KNHANES continues to expand its survey components to address emerging health issues and to provide timely national statistics that support policy development. New components are identified through multiple channels, including the KNHANES website, relevant ministries and agencies, and the national health survey steering committee. Their public health relevance, feasibility, respondent burden, and budget implications are reviewed both internally and by the advisory committee to determine whether adoption is appropriate.

In the 10th KNHANES, an in-depth survey on older adults’ health was introduced to respond to rapid population aging and the increase in single-person households (Table 2). Korea is experiencing one of the fastest rates of population aging among OECD countries [13]. Adults aged ≥65 years comprised 15.7% of the population in 2020 [14] and approximately 20% by the end of 2024, marking Korea’s transition into a super-aged society [15]. The WHO defines “healthy ageing” as the process of developing and maintaining functional ability that enables well-being in older age, emphasizing quality of life rather than the absence of disease alone [16,17]. To respond to these changes, KNHANES reinstated the osteoporosis examination in 2024. This assessment uses dual-energy X-ray absorptiometry (DXA) to measure bone mineral density at the lumbar spine and femur (total and neck), as well as whole-body fat and muscle mass. In addition, clinical markers related to osteoporosis, including serum intact parathyroid hormone and alkaline phosphatase, are analyzed. The survey will be conducted in collaboration with the Korean Society for Bone and Mineral Research. Through this initiative, KNHANES aims to monitor changes in osteoporosis prevalence and related factors since 2008–2011 and to provide evidence for establishing Korean diagnostic criteria for osteoporosis. While sarcopenia has been measured using bioelectrical impedance analysis since 2022, DXA-based estimates have been collected and released since 2024 to improve measurement accuracy. Physical function is a key indicator of quality of life among older adults, but instruments such as the activities of daily living (ADL) and instrumental activities of daily living (IADL) primarily assess disability. To capture a broader range of physical and functional abilities, including lower and upper limb function, social function, and daily performance, KNHANES developed life functioning scale and administered it to participants aged ≥65 years since 2024 [18,19]. These data enable monitoring of comprehensive functional status and evaluation of associations with aging-related conditions such as osteoporosis and sarcopenia. In the 2025 nutrition survey, new questions on the consumption frequency of major protein sources (meat, fish, eggs, beans, and dairy products) were added to assess usual protein intake and analyze its relationship with sarcopenia and osteoporosis.

Next, individual health is influenced by a wide range of social factors, including socioeconomic conditions, interpersonal relationships, and emotional circumstances. The WHO recommends monitoring social determinants of health to reduce health inequalities and to inform national health promotion policies. National health surveys in several countries, including the United States National Health Interview Survey, also routinely assess these determinants [20]. New components on social support, life satisfaction, and loneliness have been introduced in KNHANES to monitor social determinants and their associated effects on health (Table 2). The future survey cycle and implementation plan for these components will be determined based on the results of the 2025 survey.

The accelerometer-based physical activity measurement, introduced in 2024 (Table 2), was designed to address the overestimation and underestimation associated with self-reported questionnaires such as the Global Physical Activity Questionnaire and to provide a more objective and accurate assessment of physical activity; it will continue to be implemented in subsequent years.

As described earlier, additional survey components reflecting policy needs from relevant ministries were incorporated through the national health survey steering committee. Based on a policy proposal from the Ministry of Agriculture, Food and Rural Affairs, a question on participation in the food assistance program was added in the 10th KNHANES to evaluate the nutritional outcomes of the food voucher program. In 2023, health literacy was introduced in response to a proposal from the Ministry of Health and Welfare, reflecting recognition that differences in the understanding and use of health information may influence chronic disease prevention and management (Tables 1 and 2). The resulting data have been used as indicators for Health Plan 2030 and will continue to be collected in the 10th KNHANES.

To accommodate diverse data needs while minimizing respondent burden associated with the introduction of new survey components, the 10th KNHANES adopted a rotating survey system that maintains a consistent number of questions, approximately 200, each year [21]. Survey domains rotate on a 2-year or 3-year cycle, and each domain is designed to be assessed at least once within a 6-year period. Key domains related to chronic disease prevention, including smoking, alcohol consumption, physical activity, and obesity, are surveyed every 2 years, whereas mental health, quality of life, and health care utilization are surveyed every 3 years. In 2025, rotating components related to smoking, alcohol use, oral health, morbidity, injury, and safety awareness are being administered (Supplementary Material 3).

### Survey methods

To enhance respondent convenience, the 10th KNHANES has expanded the use of web-based self-administered questionnaires in both the health interview and nutrition survey. In 2020, a methodological study was conducted to facilitate the transition selected interviewer-administered components to self-administered formats, using a web-based self-administered tool [22,23]. Based on an additional expert review of these findings, components demonstrating a Kappa coefficient ≥0.6 and agreement rates ≥70% between interviewer-administered and web-based responses, including items related to activity limitation, quality of life, health care utilization, vaccination, and education, were converted to self-administered formats beginning in 2022. The eligible age range for self-administered questionnaires was gradually expanded, from adults younger than 60 years in 2022 to those younger than 70 years in 2023, and subsequently to all age groups in 2025. Since 2023, web-based self-administered surveys have been introduced while maintaining the existing paper-based option. In 2024, an additional study was conducted to evaluate the potential effects of the shift from interview-administered to self-administered survey methods. Results for items affected by these changes were compared, and no significant differences attributable to the mode of administration were observed [24]. Accordingly, the self-administered method was retained to ensure consistency while maintaining respondent comprehension. For certain items (e.g., outpatient visits, reasons for activity limitation), reference periods and explanatory notes were added to improve clarity.

In the 10th KNHANES, the nutrition survey newly introduced a web-based self-administered dietary behavior questionnaire. This instrument was developed through a research project that assessed comprehension, reliability, and validity across modes of administration [25]. The final items were confirmed through review by the nutrition subcommittee of the advisory committee. Most components demonstrated agreement rates ≥80% between face-to-face interviews and web-based responses; however, items with multiple response options, such as frequency of eating out and fruit consumption, showed lower agreement and were therefore simplified by consolidating response categories. Additional explanations and visual aids were incorporated to further improve comprehension. The dietary behavior survey includes individuals aged 1 year and older. The mode of administration varies by age to account for feasibility and differences in online device literacy. Web-based self-administered questionnaires are administered to individuals aged 12–69 years, whereas children aged 1–11 years complete the survey through proxy responses from parents or guardians. For adults aged ≥70 years, face-to-face interviewer-administered surveys serve as the default mode, with web-based completion permitted when feasible. In the health interview and nutrition surveys, participants may complete a web-based or paper-based self-administered questionnaire in advance or use a tablet PC at the MECs. When self-administration is difficult, such as among some older adults, interviewer-administered surveys are provided with on-site assistance to support participation. To enhance the reliability and validity of the survey results, field staff check for logical inconsistencies on site, and differences in results across modes of administration are reviewed by the advisory committee prior to the public release of the microdata.

As in previous cycles, the health examinations, which require direct physical measurements and the collection of blood and urine samples, continue to be conducted in person by trained professionals using standardized equipment at the MECs.

### Survey operations

Following the establishment of the Korea Disease Control and Prevention Agency (KDCA) and the creation of the Regional Centers for Disease Control and Prevention (RCDC) in 2020, responsibility for implementing the KNHANES was delegated to the RCDC. To ensure reliable survey operations, the KDCA and RCDC jointly conduct interviewer training, competency assessments, and data quality control. In the 10th KNHANES, the fundamental operational structure remains consistent with previous cycles. This structure includes year-round data collection using MECs; 4 field survey teams, each consisting of 10 staff members, for a total of 40 personnel; standardized training programs; field supervision; and systematic data quality management. The key operational procedures are summarized below.

Field survey teams undergo standardized training and periodic assessments, with feedback and individualized retraining provided as needed. Examinations such as blood pressure measurement, pulmonary function testing, and bone density assessment are conducted in collaboration with relevant academic societies. Regular site visits are used to evaluate field performance and ensure compliance with quality-control guidelines. Data cleaning is performed in accordance with standardized protocols, with routine checks conducted to ensure data accuracy. For the health examination component, on-site weekly cross-checking among field staff is performed, and the KDCA conducts weekly data reviews and biannual inter-observer and intra-observer reliability assessments. For the health interview component, 1 complete interview per interviewer is audio-recorded or video-recorded each quarter and reviewed to identify errors and provide feedback. Weekly peer cross-checking and monthly comprehensive reviews by KDCA staff are also implemented to maintain data quality. For the nutrition survey, all 24-hour dietary recall data undergo full verification, and the entire survey process is audio-recorded or video-recorded biannually to identify errors and support quality improvement.

In the 10th KNHANES, physicians dispatched by the Korean Academy of Family Medicine to the MECs, first introduced in 2024, continue to manage field operations, support survey quality, and enhance participant satisfaction. These physicians provide consultations on examination results and early-notification clinical indicators, including blood pressure, fasting glucose, triglycerides, and other laboratory test results. They also ensure appropriate responses to urgent clinical situations, thereby contributing to stable and efficient on-site survey operations.

### Data utilization

The KNHANES is a key source of data for setting and evaluating performance targets for major government health policies, including the National Health Plan, the National Nutrition Plan, and the National Oral Health Plan. It is also widely used as foundational evidence for developing chronic disease fact sheets, clinical guidelines for prevention and treatment, and the Dietary Reference Intakes for Koreans.

Survey results are published in the Health Statistics report and, together with the microdata and user guides, are publicly available on the KNHANES website (https://knhanes.kdca.go.kr/). As of 2024, there had been 151,118 cumulative microdata requests from 53,629 users, and approximately 3,700 studies using KNHANES data had been published in international journals (https://pubmed.ncbi.nlm.nih.gov). In addition, topic-specific analytical reports, *Health Statistics Plus*, have been published regularly since 2021 to support health policy planning and evaluation.

To increase the utility of the survey, linked databases integrating data from other government agencies, including Statistics Korea and the Ministry of Environment, have been developed since 2007 and made publicly available since 2021. For the KNHANES–Causes of Death Statistics linkage, data from 69,855 adults aged ≥19 years who participated in the 2007–2018 KNHANES, with a linkage rate of 97.5%, were linked to the 2007–2023 Causes of Death Statistics and released in the first half of 2025. Going forward, KNHANES data will be updated every 3 years and mortality data annually; in 2026, data from KNHANES 2007–2021 will be linked to the 2024 Causes of Death Statistics. Air Quality Data from the Ministry of Environment are also linked for 114,568 participants from the 2007–2022 KNHANES cycles using daily modeled air pollution concentrations from 2005–2022, with a linkage rate of 100% [26-30]. Although the cancer registry of the National Cancer Center and datasets from the National Health Insurance Service are not publicly released, KNHANES-linked data are accessible through the Health and Medical Big Data Platform. Between 2018 and 2024, 83 of 95 provisions on the platform, representing 87%, involved KNHANES-linked datasets, demonstrating their high utility. The KNHANES has collected and stored biospecimens from participants since 2007. As of September 2025, biospecimens and linked clinical and epidemiological data from 41,737 participants, collected between 2013 and 2023, had been distributed for research. These resources have been used in studies on chronic diseases, air pollution–related lung function, and infectious diseases. Researchers may request biospecimens through the National Biobank of Korea (https://biobank.nih.go.kr) and link them to KNHANES epidemiological data. To enable the use of KNHANES data in conjunction with health and medical information for generating evidence to support chronic disease prevention and management and to better respond to policy needs, a legal basis, Article 30-2 of the National Health Promotion Act, was established in 2025.

### Korea National Health and Nutrition Examination Survey-Follow-up

One of the major new features of the 10th KNHANES is the introduction of a longitudinal follow-up survey, the KNHANES Follow-up Survey (KNHANES-F), designed to identify factors related to the onset and progression of chronic diseases.

Given the trend toward earlier onset of chronic diseases in Korea, it is important to monitor changes in health behaviors and health status among children, adolescents, and young adults to establish evidence for the early prevention and management of chronic disease incidence and progression. However, most ongoing chronic disease follow-up surveys in Korea, including those conducted by the KDCA, primarily target middle-aged and older adults [31]. Therefore, KNHANES-F was designed to include individuals aged 10–59 years, a population group with limited representation in previous surveys, while also considering the feasibility of web-based self-administered participation (Table 3). Planning for KNHANES-F was completed in 2023, and survey components and target diseases were selected by age group in 2024 [32]. The survey was launched in 2025.

Participants include individuals aged 10–59 years who take part in the 10th KNHANES and provide consent for follow-up. The target consent rate is 70%, with an expected 8,000–9,000 participants enrolled over 3 years. KNHANES-F consists of a baseline survey conducted in the first year of participation and annual follow-up surveys extending for more than 10 years. Target diseases were selected based on disease burden, epidemiologic trends, and the need for follow-up data not covered by other cohort studies or linked datasets. Adult target diseases include 19 conditions, such as diabetes, hypertension, cancer, and depression. For children and adolescents, 8 conditions were selected, including depression, precocious puberty, and asthma.

The baseline survey, conducted from 2025 to 2027, excludes components duplicated in the main KNHANES to minimize respondent burden and includes items such as birth weight, age at smoking initiation, and age at chronic disease diagnosis. The baseline questionnaire contains 40 items for children, 53 for adolescents, and 45 for adults, as well as a 168-item web-based food frequency questionnaire for adolescents and adults with validated reliability and validity [25]. Follow-up survey components were selected based on indicators requiring repeated measurement and include additional items such as reproductive health indicators. The follow-up questionnaire contains apporoximately 100 items for children, 300 items for adolescents, and 400 items for adults. Core components are assessed annually, while rotating components follow a 3-year cycle. Common components across all age groups include household information, morbidity, smoking, physical activity, sleep, mental health, and women’s health. For children and adolescents, components include growth and development, precocious puberty, and attention deficit hyperactivity disorder. Adult components include chronic disease diagnosis and management and quality of life. Survey components may be refined based on recommendations from the advisory committee. KNHANES-F is conducted entirely through web-based self-administered questionnaires, with data quality monitored for missing or inconsistent responses and supplemented by telephone follow-up when necessary. To maintain follow-up rates, regular participant contact, periodic health information updates, and participation incentives are provided.

### International collaboration

The KNHANES microdata have been continuously used in international collaborative studies on chronic diseases. Representative examples include the provision of data to the NCD Risk Factor Collaboration since 2015 and to the Global Dietary Database Consortium since 2019, in which Korea participates as an active research partner [33-36].

In April 2025, the KDCA was designated as a WHO Collaborating Centre for NCD Surveillance and Big Data Utilization. As the first collaborative activity under this designation, the KDCA co-organized the Regional Workshop on Translating Data into Policy Action for NCD Prevention with the WHO Western Pacific Regional Office in October 2025. During the workshop, the KDCA shared operational experiences from the 10th KNHANES, including survey contents and methods and the introduction of the KNHANES-F, with participating Member States. Over the next 4 years, the WHO Collaborating Centre will undertake 4 areas of work: strengthening surveillance capacity for evidence-based NCD prevention and management, supporting the establishment and operation of NCD survey systems, enhancing health facility–based NCD surveillance, and improving analytical capacity for the utilization of health big data (Table 4).

## CONCLUSION

The 10th KNHANES aims to comprehensively assess aging-related health indicators, including sarcopenia, osteoporosis prevalence and management, and life functioning, to better understand the health challenges associated with Korea’s transition to a super-aged society. These data are expected to provide essential evidence for planning tailored health promotion programs for older adults, setting policy priorities, and supporting related research. Survey methods have also been progressively refined, with the dietary behavior questionnaire and food frequency questionnaire in the nutrition survey transitioning to web-based self-administered formats.

Moving forward, KNHANES will continue to produce timely health indicators essential for national health policy development and academic research. With the establishment of a legal basis for data linkage, additional data sources from external institutions will be identified, and the scope of linked datasets and public access will be further expanded. Longitudinal follow-up data will be used to examine changes in health determinants and their associations with chronic disease outcomes. Through the operation of the WHO Collaborating Centre, KNHANES will further strengthen its role as a national health surveillance system by sharing chronic disease monitoring frameworks and policy outcomes with the international community.

### Ethics statement

This study was approved by the Institutional Review Board of the KDCA (2025: IRB No. 2022-11-16-7C-A).

## Figures and Tables

**Table 1.** Summary of the main features of the 10th KNHANES (2025–2027)

Category	Contents	Effects
Survey population	192 primary sampling units and 4,800 households annually	Maintained statistical representativeness
Survey contents	Aging: Osteoporosis, sarcopenia, life functioning scale, vitamin D, frequency of intake of meat, fish, eggs, and beans, frequency of intake of milk and dairy products	Strengthens the evidence base for health policies, including emerging health issues
	Social determinants of health: Social support	
	Health policy: Health literacy, food assistance program	
Survey methods	Health interview: Expansion of web-based self-administered surveys to all age groups	Enhances survey convenience
	Nutrition: Introduction of web-based self-administered surveys	
Survey operations	Establishment of physician-led supervision and on-site oversight	Improves survey quality control
KNHANES-Follow-up Survey	Introduction of follow-up surveys	Enhances evidence for policy on chronic disease prevention and management
International collaboration	Designated as a WHO Collaborating Centre for NCD Surveillance and Big Data Utilization	Strengthens international collaboration in chronic disease surveillance

KNHANES, Korea National Health and Nutrition Examination Survey; WHO, World Health Organization; NCD, non-communicable diseases.

**Table 2.** Survey components of the 10th Korea National Health and Nutrition Examination Survey (2025-2027)

Survey components	Eligible age (yr)	Description
Health examination		
Anthropometry (body measures)	1+	Height, weight, waist (6 and older)
Blood pressure	6+	Systolic and diastolic blood pressure, pulse rate
Venipuncture and urine	10+	Diabetes: Blood glucose, hemoglobin A1c
		Dyslipidemia: Total cholesterol, LDL-cholesterol, HDL-cholesterol, triglycerides
		Kidney disease: Creatinine, microalbumin (urine)
		Liver disease: Hepatitis C surface antigen, hepatitis C antibody, hepatitis C RNA, aspartate aminotransferase, alanine aminotransferase
		Anemia: Hemoglobin, hematocrit
		Osteoporosis: Vitamin D, intact parathyroid hormone, alkaline phosphatase (19 and older)
		CBC: Red blood cell count, white blood cell count, platelets, etc.
Body composition	65+	Lean mass, fat mass, total body water
Grip strength test	65+	Grip strength
Autorefraction	10–39 (2025)	Refractive error
	40–59 (2026)	
	60+ (2027)	
Dual-energy X-ray absorptiometry	19+	Bone mineral contents, bone mineral density, lean mass, fat mass
Spirometry	40+	Forced vital capacity, forced expiratory volume in 1 sec
Health interview		
Household survey	19+	Marital status, no. of household members, type of household, housing ownership status, housing type, household income, health insurance, private insurance, public assistance recipient status
Education	1+	Education level, graduation status
	19–64	Parental education level
Economic activity	15+	Employment status, type of employment, job status classification, job type, total working hours
	19+	Reason for unemployment, permanent employment status, work schedule type, (longest-held job) job type^1^, type of employment^1^, job status classification^1^
Disease history	1+	Self-rated health status, illness in the past 2 wk, disease history
	19+	Disease history, family history
Medical utilization	1+	Unmet medical needs/reason for unmet medical needs, outpatient visit, hospitalization experience
Health literacy	19+	Health literacy
Health checkup	19+	Health checkup participation, cancer screening
Vaccination	1+	Influenza vaccination
Activity limitation, quality of life	12+	Monthly absence
	19+	Activity limitation status/reason, experience of illness, absence from work or school in the past mo/no. of days, EQ-5D, social support^2^, life satisfaction^2^
	65+	Life functioning scale
Injury	1+	Injury experience, no. of occurrences, time of occurrence, place of treatment for injury, bed rest due to injury, no. of days confined to bed due to injury, absence from work or school due to injury, no. of days absent from work/school due to injury
Smoking	12–18	Smoking experience, age at first smoking, current smoking status, amount of smoking
	19+	Cigarettes smoking experience /age started daily smoking/amount of smoking, heated tobacco product use experience/age started daily use/amount of use, E-cigarette use experience/age started daily use, other tobacco products use experience, age of first smoking/types of tobacco products, quit attempt/plan to quit smoking, secondhand smoke exposure at home· workplace indoor public paces
Alcohol use	12+	Lifetime consumed alcohol, age at first drinking, frequency of drinking, drinking amount, frequency of binge drinking, alcohol-related harm
	19+	Advice to reduce alcohol consumption, experience of counseling for alcohol related problems
Physical activity	12–18	Engagement in 60 min of physical activity per day, sedentary time, muscle strengthening exercise
	19+	(Work/leisure) vigorous/moderate physical activity, activity during transportation, sedentary time, walking, muscle strengthening exercise
	19–64	Measurement of physical activity level (using an accelerometer)
Sleep health	12+	Wake-up and bedtime (weekdays/weekends)
Mental health	12–18	Suicidal plans
	12+	Perceived stress level, experience of depressive, experience of suicidal thoughts/attempts, experience of mental health counseling
	19+	PHQ-9, experience of loneliness^2^
Safety awareness	19+	Experience of driving a bicycle, motorcycle, or car under the influence of alcohol, riding with a drunk driver
Obesity, weight control	6+	Perception of body shape, efforts to control weight
Women’s health	10+	Current menstruation status, age at first menstruation
	15+	Pregnancy experience/no. of pregnancies, childbirth experience/age at childbirth
	19+	Age at menopause, breastfeeding experience/no. of children breastfed and duration, experience of oral contraceptive use/total duration of use cause of artificial menopause, experience of hormone therapy/age at first use/total duration use
Oral health	1+	tooth brushing practice/timing, reason for tooth damage, oral checkup, dental service use/treatment items, unmet dental care needs/reasons
	12+	Use of hygiene products
	19+	Chewing difficulty, speech difficulty
Nutrition survey		
Dietary habits	1+	Breakfast frequency, frequency of restaurant food consumption, use of dietary supplements, dietary control status and reason, average daily water intake, frequency of consumption for fruit, meat, fish, eggs, milk and milk products and beans
	12+	Experience of nutrition education or counseling, awareness and use for nutrition labels
	1–3	Breast feeding and formula feeding, timing of complementary feeding
Household food security	Household (14+)	Household food security, household food assistance program participation and type
24-hr recall	1+	Foods and amounts consumed during the day the two days before the survey
Household food preparer	Household food preparer	Information on food ingredients in household-prepared foods consumed during the day the two days before the survey

LDL, low-density lipoprotein; HDL, high-density lipoprotein; CBC, complete blood count; EQ-5D, EuroQol-5 Dimension; PHQ-9, Patient Health Questionnaire 9.

1 A survey targeting economically inactive individuals aged 19–59 years or those aged 60 years and older.

2 Survey items may be modified through revisions to the survey plan.

**Table 3.** Overview of the Korea National Health and Nutrition Examination Survey-Follow-up

Category	Contents	
Survey population	Individuals aged 10–59 yr who participated in the 10th KNHANES (2025–2027) and agreed to follow-up	
Survey method^1^	Web-based self-administered questionnaire via smartphone, PC, or tablet PC In the case of children aged 10–11 yr, guardians responded on their behalf	
Survey schedules	Baseline	Once in the year of consent to participate in the survey
	Follow-up	Conducted annually after the baseline survey (with a target duration of 10 yr or more)
Survey components	The questionnaires are differentiated by age groups	
	Children (10–11 yr), adolescents (12–18 yr), and adults (≥19 yr)	
	Children	Household survey, morbidity, smoking, physical activity, sleep, mental health, women’s health, growth and pubertal development, etc.
	Adolescents	Household survey, education, economic activity, morbidity, smoking, alcohol use, physical activity, sleep, mental health, social support, obesity and weight control, women’s health, growth and pubertal development, FFQ, etc.
	Adults	Household survey, education, economic activity, socioeconomic status, morbidity, quality of life, smoking, alcohol use, physical activity, sleep, mental health, social support, obesity and weight control, women’s health, dietary behavior, FFQ, etc.
Survey cycle	Annual (core, 1-yr cycle)	Household survey, quality of life, smoking, alcohol use, physical activity, dietary behavior, etc.
	Triennial (rotating, 3-yr cycle)	Morbidity, social support, women’s health, FFQ, etc.

KNHANES, Korea National Health and Nutrition Examination Survey; FFQ, food frequency questionnaire.

1 Survey components may be modified through revisions to the survey plan.

**Table 4.** Terms of reference and activities of the WHO Collaborating Centre for NCD Surveillance and Big Data Utilization

Category	Contents
TOR 1	Support WHO’s activities on providing technical support to Member States in the Western Pacific for improving the utilization of NCD data for informing evidence-based policies and programs at the country level
Activity	At WHO’s request, provide technical support to WHO’s activities on organizing a “Data to Action” workshop to build capacity for Member States in the area of utilizing data for actions and decisions for NCD prevention and control
TOR 2	Support WHO’s activities on providing technical support in the area of data collection analysis and utilization, tailored for specific needs of at least one country
Activity	At WHO’s request, provide technical support to WHO`s activities on building capacity in big data utilization related to NCDs
TOR 3	Support WHO’s activities on providing technical support to Member States in strengthening facility-based surveillance of NCDs
Activity	At WHO’s request, develop workshop materials that may inform its activities when conducting in-country trainings to support the development of facility- based surveillance of NCDs such as hypertension, cardiovascular disease, diabetes and cancer
TOR 4	Support WHO’s activities on strengthening capacity of Member States in health-related big data analysis techniques
Activity	At WHO’s request, provide technical support to WHO’s activities on building capacity in big data analysis related to mortality, priority communicable and NCDs

WHO, World Health Organization; NCD, Non-communicable disease; TOR, terms of reference.

## Supplementary materials

Supplementary Material 1. Floor plan of the Mobile Examination Center (MEC) in the 10th Korea National Health and Nutrition Examination Survey (2025-2027)

Supplementary Material 2. Sample design in the 10th Korea National Health and Nutrition Examination Survey (2025-2027)

Supplementary Material 3. Rotating survey system for the health interview in the Korea National Health and Nutrition Examination Survey
