# Supplementary material for: Plan and operations of the 10th Korea National Health and Nutrition Examination Survey (2025–2027)
Source: Epidemiol Health. 2026 Jan 2;48:e2026001. doi: 10.4178/epih.e2026001 (PMC12946570; doi:10.4178/epih.e2026001)
Supplement: Supplementary Material 1. — Floor plan of the Mobile Examination Center (MEC) in the 10th Korea National Health and Nutrition Examination Survey (2025-2027) [file epih-48-e2026001-Supplementary-1.docx]

**Supplementary Material 1.** Floor plan of the Mobile Examination Center (MEC) in the 10th Korea National Health and Nutrition Examination Survey (2025-2027)

**- Blood draw**

**- Urine collection**

**Health Interviews**

**Autorefraction**

**- Pulmonary function test**

**- Grip strength**

**- Blood pressure**

**- Body measures**

**- Bioelectrical Impedance**

**analysis**

**Dental examination**

**Bone density**

**Dietary**

**interviews**

**Dietary**

**interviews**

**Physician**

**Health Interviews**

**Changing Room**

**Changing Room**

**Reception**
