# Supplementary material for: Plan and operations of the 10th Korea National Health and Nutrition Examination Survey (2025–2027)
Source: Epidemiol Health. 2026 Jan 2;48:e2026001. doi: 10.4178/epih.e2026001 (PMC12946570; doi:10.4178/epih.e2026001)
Supplement: Supplementary Material 2. — Sample design in the 10th Korea National Health and Nutrition Examination Survey (2025-2027) [file epih-48-e2026001-Supplementary-2.docx]

**Supplementary Material 2.** Sample design in the 10th Korea National Health and Nutrition Examination Survey (2025-2027)

|  | **No. of Sampling units in the 10^th^**  **KNHANES (2025-2027)** | | | | | | | | |  | **No. of Sampling units by year**^1)^ | | | | | | | | | |
| --- | --- | --- | --- | --- | --- | --- | --- | --- | --- | --- | --- | --- | --- | --- | --- | --- | --- | --- | --- | --- |
|  | Housing type | | | | | | | | |  | Housing type | | | | | | | | | |
|  | Total | Non-apartment | | | | Apartment | | | |  | Total | Non-apartment | | | | Apartment | | | | |
|  |  | Dong | | Eup·Myeon | | Dong | | Eup·Myeon | |  |  | Dong | | Eup·Myeon | | Dong | | Eup·Myeon | |  |
| **Total** | 576 | | 216 | | 63 | | 264 | | 33 |  | 192 | | 72 | | 21 | | 88 | | 11 |  |
| **Province** |  | |  | |  | |  | |  |  |  | |  | |  | |  | |  |  |
| Seoul | 102 | | 57 | | - | | 45 | | - |  | 34 | | 19 | | - | | 15 | | - |  |
| Busan | 36 | | 15 | | - | | 21 | | - |  | 12 | | 5 | | - | | 7 | | - |  |
| Daegu | 27 | | 12 | | - | | 12 | | 3 |  | 9 | | 4 | | - | | 4 | | 1 |  |
| Incheon | 33 | | 15 | | - | | 18 | | - |  | 11 | | 5 | | - | | 6 | | - |  |
| Gwangju | 18 | | 6 | | - | | 12 | | - |  | 6 | | 2 | | - | | 4 | | - |  |
| Daejeon | 15 | | 6 | | - | | 9 | | - |  | 5 | | 2 | | - | | 3 | | - |  |
| Ulsan | 18 | | 6 | | 3 | | 6 | | 3 |  | 6 | | 2 | | 1 | | 2 | | 1 |  |
| Sejong | 12 | | 6 | | - | | 6 | | - |  | 4 | | 2 | | - | | 2 | | - |  |
| Gyeonggi | 132 | | 42 | | 15 | | 66 | | 9 |  | 44 | | 14 | | 5 | | 22 | | 3 |  |
| Gangwon | 18 | | 6 | | 3 | | 9 | | - |  | 6 | | 2 | | 1 | | 3 | | - |  |
| Chungbuk | 18 | | 6 | | 3 | | 9 | | - |  | 6 | | 2 | | 1 | | 3 | | - |  |
| Chungnam | 24 | | 6 | | 6 | | 9 | | 3 |  | 8 | | 2 | | 2 | | 3 | | 1 |  |
| Jeonbuk | 21 | | 6 | | 6 | | 9 | | - |  | 7 | | 2 | | 2 | | 3 | | - |  |
| Jeonnam | 21 | | 6 | | 6 | | 9 | | - |  | 7 | | 2 | | 2 | | 3 | | - |  |
| Gyeongbuk | 33 | | 9 | | 9 | | 9 | | 6 |  | 11 | | 3 | | 3 | | 3 | | 2 |  |
| Gyeongnam | 36 | | 9 | | 9 | | 12 | | 6 |  | 12 | | 3 | | 3 | | 4 | | 2 |  |
| Jeju | 12 | | 3 | | 3 | | 3 | | 3 |  | 4 | | 1 | | 1 | | 1 | | 1 |  |

1) Sampling units were consistently assigned across 2025, 2026, and 2027, with 192 units each year.
