# Supplementary material for: Plan and operations of the 10th Korea National Health and Nutrition Examination Survey (2025–2027)
Source: Epidemiol Health. 2026 Jan 2;48:e2026001. doi: 10.4178/epih.e2026001 (PMC12946570; doi:10.4178/epih.e2026001)
Supplement: Supplementary Material 3. — Rotating survey system for the health interview in the Korea National Health and Nutrition Examination Survey [file epih-48-e2026001-Supplementary-3.docx]

**Supplementary Material 3.** Rotating survey system for the health interview in the Korea National Health and Nutrition Examination Survey

|  |  | **2025** | **2026** | **2027** | **2028** | **2029** | **2030** |  |
| --- | --- | --- | --- | --- | --- | --- | --- | --- |
| **Core**  (Annually) |  | **All items** (Socioeconomic status, Health behaviors, Chronic diseases, Diet and nutrition, etc.) | | | | | |  |
|  |  |  |  |  |  |  |  |  |
| **Rotating**  (2-year or  3-year  cycle) |  | Injury,  Safety Awareness | Sleep health,  Mental health,  Quality of life, Women’s health | Medical Utilization, Health checkup, Vaccination | Injury,  Safety Awareness | Sleep health,  Mental health,  Quality of life, Women’s health | Medical Utilization, Health checkup, Vaccination |  |
|  |  |  |  |  |  |  |  |  |
|  |  | Smoking,  Alcohol use,  Oral health | Physical Activity,  Obesity&  Weight control | Smoking,  Alcohol use,  Oral health | Physical Activity,  Obesity&  Weight control | Smoking,  Alcohol use,  Oral health | Physical Activity,  Obesity&  Weight control |  |
|  |  |  | | | | | |  |
|  |  | Disease History | - | Disease History | - | Disease History | - |  |
| **Emerging**  **issues** |  | **New topics or items** including emerging health issues | | | | | |  |
